# Effects of tDCS on Language Recovery in Post-Stroke Aphasia: A Pilot Study Investigating Clinical Parameters and White Matter Change with Diffusion Imaging

**DOI:** 10.3390/brainsci11101277

**Published:** 2021-09-26

**Authors:** Radwa K. Soliman, Chantal M. W. Tax, Noha Abo-Elfetoh, Ahmed A. Karim, Ayda Youssef, Doaa Kamal, Eman M. Khedr

**Affiliations:** 1 Department of Diagnostic and Interventional Radiology, Assiut University Hospitals, Assiut 71515, Egypt; 2 Cardiff University Brain Research Imaging Center (CUBRIC), School of Physics and Astronomy, Cardiff University, Cardiff CF24 4HQ, UK; c.m.w.tax@umcutrecht.nl; 3 Department of Neurology and Psychiatry, Assiut University Hospitals, Assiut 71515, Egypt; noha_ahmed_re@yahoo.com (N.A.-E.); emankhedr99@yahoo.com (E.M.K.); 4 Department of Psychiatry and Psychotherapy, University of Tübingen, 72076 Tübingen, Germany; ahmed.karim@uni-tuebingen.de; 5 Department of Neuroscience, Jacobs University in Bremen, 28795 Bremen, Germany; 6 Department of Health Psychology and Neurorehabilitation, SRH Mobile University, 88499 Riedlingen, Germany; 7 Department of Diagnostic and Interventional Radiology, National Cancer Institute, Cairo University, Cairo 11796, Egypt; dr.aydayoussef@gmail.com; 8 Department of Rheumatology and Rehabilitation, Assiut University Hospitals, Assiut 71515, Egypt; doaakamal@aun.edu.eg; 9 Department of Neurology and Psychiatry, Aswan University Hospitals, Aswan 81528, Egypt

**Keywords:** diffusion imaging, constrained spherical deconvolution, white matter, transcranial direct current stimulation, aphasia recovery, frontal aslant tract, uncinate fasciculus

## Abstract

**Objectives:** In this pilot study we investigated the effects of transcranial direct current stimulation (tDCS) on language recovery in the subacute stage of post-stroke aphasia using clinical parameters and diffusion imaging with constrained spherical deconvolution-based tractography. **Methods:** The study included 21 patients with subacute post-stroke aphasia. Patients were randomly classified into two groups with a ratio of 2:1 to receive real tDCS or sham tDCS as placebo control. Patients received 10 sessions (5/week) bi-hemispheric tDCS treatments over the left affected Broca’s area (anodal electrode) and over the right unaffected Broca’s area (cathodal stimulation). Aphasia score was assessed clinically using the language section of the Hemispheric Stroke Scale (HSS) before and after treatment sessions. Diffusion imaging and tractography were performed for seven patients of the real group, both before and after the 10th session. Dissection of language-related white matter tracts was achieved, and diffusion measures were extracted. A paired Student’s *t*-test was used to compare the clinical recovery and diffusion measures of the dissected tracts both pre- and post- treatment. The partial correlation between changes in diffusion measures and the language improvements was calculated. **Results:** At baseline assessment, there were no significant differences between groups in demographic and clinical HSS language score. No significant clinical recovery in HSS was evident in the sham group. However, significant improvements in the different components of HSS were only observed in patients receiving real tDCS. Associated significant increase in the fractional anisotropy of the right uncinate fasciculus and a significant reduction in the mean diffusivity of the right frontal aslant tract were reported. A significant positive correlation was found between the changes in the right uncinate fasciculus and fluency improvement. **Conclusions:** Aphasia recovery after bi-hemispheric transcranial direct current stimulation was associated with contralesional right-sided white matter changes at the subacute stage. These changes probably reflect neuroplasticity that could contribute to the recovery. Both the right uncinate fasciculus and right frontal aslant tract seem to be involved in aphasia recovery.

## 1. Introduction

Aphasia is one of the most common catastrophic post-stroke consequences. Most aphasic patients, however, retain a degree of functional recovery within few weeks or months after stroke [1]. The degree of language recovery can be augmented by modulating brain activity using non-invasive brain stimulation tools, e.g., transcranial direct current stimulation (tDCS) [2]. Previous reports revealed that tDCS has a positive impact on aphasia rehabilitation [2]. tDCS uses a constant, low intensity current delivered via two or more electrodes placed over the head, which can modulate neuronal activity. Previous studies have demonstrated that cerebral excitability was diminished by cathodal stimulation, which hyperpolarizes neurons, whereas anodal stimulation depolarizes neurons and increases cerebral excitability [3,4,5,6]. In neurorehabilitation studies, the anodal electrode is usually placed over the target area to induce excitatory changes and increase brain activity. In other cases, aiming to induce bilateral modulation, the cathodal electrode is placed over the contralateral area inducing inhibitory changes and thereby suppressing maladaptive activity [2]. Remarkably, Bolzoni et al. [7] found that tDCS in anesthetized animals not only affects cortical neurons, but also facilitates activation of neurons in subcortical motor systems. Moreover, subcortical facilitation outlasts the time duration of transcranial stimulation. These findings provide intriguing evidence for tDCS induced neuroplasticity at subcortical levels. Zheng and Schlaug [8] found that structural white matter changes in descending motor tracts using diffusion tensor imaging correlate with improvements in motor impairment after undergoing a treatment course of tDCS and physical therapy. Previous functional imaging studies provided evidence of neuroplasticity changes in the language areas following aphasia therapy in stroke survivors [9,10]. However, only a few reports have investigated the underlying white matter (WM) changes following aphasia therapy [11,12,13,14,15,16], (mainly after language training or melodic intonation therapy). These studies employed diffusion tensor Imaging (DTI), where diffusion measures are derived to quantify microstructural organization of WM tracts. Fractional anisotropy (FA) and mean diffusivity (MD) were the most commonly used measures. While FA reflects the degree of anisotropy, MD is sensitive to barriers of diffusion in all directions within the voxel [17]. These measures have been correlated with a number of structural features such as axonal density, thickness of myelin, as well as coherence of fibers [18].

The above-mentioned studies reveal that changes in white matter properties were associated with aphasia improvement. It is thought that such effects represent a form of anatomical plasticity caused by sprouting of new connections and restitution of axonal function that are necessary for clinical improvement. However, given its important role in language function, previous studies focused mainly on the dorsal stream represented by the arcuate fasciculus (AF), with only very few of them including the ventral stream, evaluating either the inferior longitudinal fasciculus (ILF) [12] or the uncinate fasciculus (UF) [14,16]. None of the previous studies evaluated the frontal aslant tract (FAT), a relatively recently identified tract that has been related to fluency [19]. Other postmortem anatomical and electrophysiological reports supported its existence and its role in language processing [20,21]. Recently Zhao et al. [22] investigated whether WM integrity, measured by DTI, predicts language therapy effects with and without tDCS over the left inferior frontal gyrus (IFG) in primary progressive aphasia. They found that the improvement in the tDCS group was greater and generalized to untrained words. White matter integrity of ventral language pathways predicted tDCS effects in trained items whereas white matter integrity of dorsal language pathways predicted tDCS effects in untrained items. They concluded that white matter integrity influences both language therapy and tDCS effects. Their data suggest that tDCS may help to circumvent white matter damage in neurodegenerative disorders, probably by modulating functional connectivity. However, to the best of our knowledge, no previous study evaluated the effect of tDCS on WM changes in post stroke aphasia. We hypothesized that (tDCS) can improve language recovery in the subacute stage of post-stroke aphasia using clinical parameters compared to a control group receiving sham tDCS as placebo control. Moreover, we hypothesized that clinical improvement will be associated with WM changes that can occur either in the ipsilateral or contralateral language related WM tracts as a compensatory mechanism for aphasia recovery.

## 2. Materials and Methods

### 2.1. Patients

**Figure 1:**
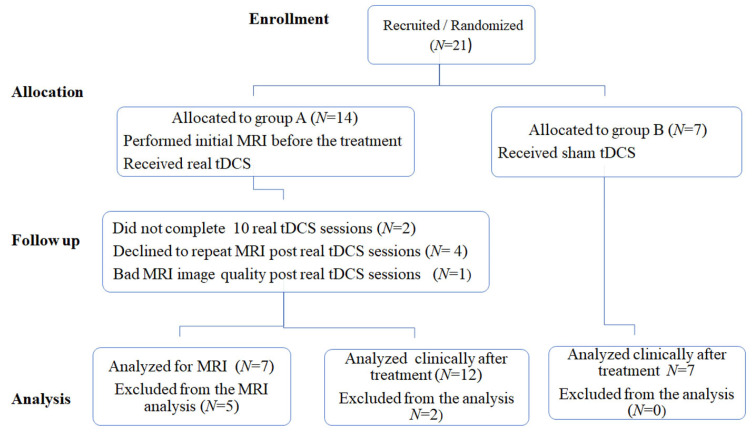
Flow chart showing the distribution of the studied groups and the follow up sessions.

Twenty-one right-handed patients with post-stroke non-fluent aphasia, at the sub-acute stage, were recruited consecutively during the period of September 2020 to March 2021 in this study. All patients were admitted to the Stroke Unit, Department of Neurology, Assiut University Hospital, Assiut, Egypt. Each patient fulfilled the inclusion criteria as follows: subacute hemiplegia (within the end of 1st to 5th weeks of onset) with non-fluent aphasia, single thromboembolic infarction documented by CT brain in the distribution of middle cerebral artery. Age ranged from 32–68 years at onset. Exclusion criteria included head injury or neurological disease other than stroke; unstable cardiac dysrhythmia; fever; infection; hyperglycemia, and prior administration of tranquilizer; patients with any contraindication to MRI were also excluded. The patients were randomly classified into two groups with a ratio of 2:1 to receive real tDCS in the experimental group (14 patients) or sham tDCS (7 patients), as a placebo condition in the control group (cf. Figure 1: flow chart). Clinical assessments were performed both pre and post the 10th tDCS session in the experimental and the control group. MRI scans were performed both pre- and post-tDCS sessions for patients receiving real tDCS but not for patients receiving sham tDCS. All patients were native Arabic speakers. Two patients refused to complete the 10 sessions as they came from a faraway area, and another patient had bad quality MRI, all of them from active group. The mean age ± SD of each group was 52.58 ± 13.12 ranging from 32–68 years and 53.71 ± 6.89 ranging from 45–61 years for the real and sham group, respectively with a male/female ratio of 8/4, and 5/2 for the real and sham group, respectively.

Written informed consent was obtained from first degree relatives, and the protocol was approved by Assiut Medical School Ethical Review Board with the IRB local approval number 17300657.

### 2.2. Clinical Assessment

A detailed case history and neurological examination was performed for each patient. The language section of the Hemispheric Stroke Scale (HSS) [23] was used in Arabic to evaluate the aphasia score for each patient. The scale encompasses four elements, i.e., comprehension, naming, repetition, and fluency, (a score of 20 represents the worst overall speech status while a score of zero represents normal speech).

For language comprehension, the patients were given three commands: “Stick out your tongue” or “Close your eyes” “Point to the door” “Place left/right hand on left/right ear and then on left/right knee (using unaffected side)”. For naming, the patients were asked to name the following items: watch or belt; watch strap or belt buckle; index finger or ring finger. For repetition, the patients were asked to repeat the following: a single word, such as “dog” or “cat”; “The president lives in Egypt”; “No ifs, ands, or buts”. For fluency, the patients were asked to name as many words as they could beginning with the letter A (i.e., the first letter of the Arabic alphabet) within one minute.

### 2.3. tDSC Procedure

**Figure 2:**
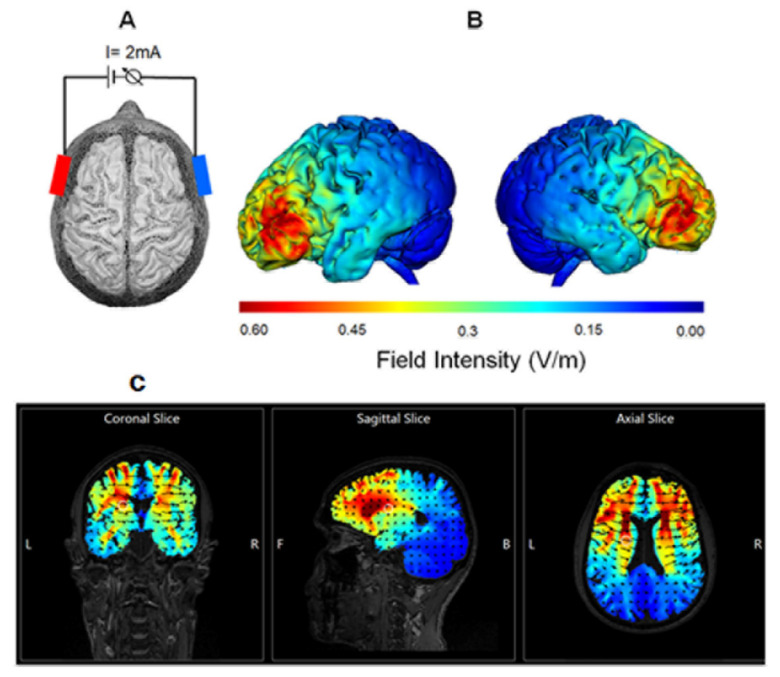
Panel (**A**) illustrates the technique used for transcranial direct current stimulation (tDCS). tDCS was delivered using a current intensity of 2 mA, and the stimulation duration was 20 min. The anodal electrode (red, 24 cm^2^) was placed over the left Broca’s area, while the cathodal electrode (blue, 24 cm^2^) was placed over the right homologue of Broca’s area. Panel (**B**,**C**) reveal the field intensity distribution in the present study according to computational simulation.

tDCS was delivered using a current intensity of 2 mA, and the stimulation duration was 20 min. Each patient received five daily sessions/week for two consecutive weeks. A pair of 24-cm^2^ rubber electrodes (neuro Conn, Ilmenau, Germany) were inserted into sponge pads soaked with 10 mL of sterile water. These were placed on the scalp according to the coordinates used by Gough et al. [24] covering the pars triangularis and opercularis. (Pars triangularis was 2.5 cm posterior to the canthus along the canther–tragus line and 3 cm superior to this line; the Pars opercularis was 4.5 cm posterior and 6 cm superior to the canther–tragus line). The anodal electrode was placed over the left over Broca’s area, while the cathodal electrode was placed over the right homologue of Broca’s area. For sham tDCS, the placement of the electrodes, current intensity, and ramp time was identical to the atDCS stimulation group; however, the stimulation only lasted for 30 s. The rationale behind this sham procedure was to mimic the transient skin sensation at the beginning of real tDCS without producing any conditioning effects on the brain. This method of sham stimulation has been shown to be reliable [25]. The physician responsible for delivering tDCS had no contact with the patients. We estimated the resulting electrical field intensity in the present study using the HD Explore software (Soterix Medical, New York, NY, USA; cf. [26], (Figure 2).

### 2.4. MRI Examination

#### 2.4.1. MRI Protocol

All subjects underwent MRI examination using a superconducting 1.5 Tesla unit (Phillips, Achieva, Best, The Netherlands). Diffusion MRI data were acquired in 3D plane by using a single shot echo planner sequence with, voxel size 2 × 2 × 2 and matrix size = 128 × 128 × 60, at 32 directions with a b-value = 1000 s/mm^2^ and single b = 0 s/mm^2^ image. 3D T1 weighted images were also acquired with voxel size = 1 × 1 × 1 m, slice thickness = 2 mm and matrix size of 208 × 208. Four patients from the experimental group declined to undergo a repeat MRI scan after completing the sessions (cf. flowchart in Figure 1).

#### 2.4.2. Imaging Processing and Analysis

##### Processing of T1-WI Data

For the patient group receiving real tDCS, normalization of T1-weighted images to standard MNI space was performed with SPM-12 (http://www.fil.ion.ucl.ac.uk/spm, accessed on 20 September 2021). Using MRIcron (www.mccauslandcenter.sc.edu/mricro/mricron, accessed on 20 September 2021), lesions were manually demarcated on T1-weighted images and the lesion volume for each patient was calculated. The segmented lesions were overlaid together on a template T1-weighted image to obtain the lesion overlay map. The map reveals the regions with least and highest lesion overlap percentage.

##### Processing of Diffusion Data

The quality of diffusion images was visually assessed for all patients, and one patient had to be excluded because of the low quality of the dataset at the post-tDCS scan. Processing of the diffusion MRI images was performed using Explore DTI (www.exploredti.org, accessed on 20 September 2021) [27]. At the outset, correction of Gibbs ringing artifact was performed followed by correction of eddy current and motion artifacts [28,29]. Correction of EPI distortion along with non-rigid registration to the 3D T1weighted image was then achieved. The diffusion tensor was then fitted with a weighted linear least square approach [30]. Eventually, whole-brain tractography, via constrained spherical deconvolution (CSD) with recursive calibration, was obtained [31,32].

**Figure 3:**
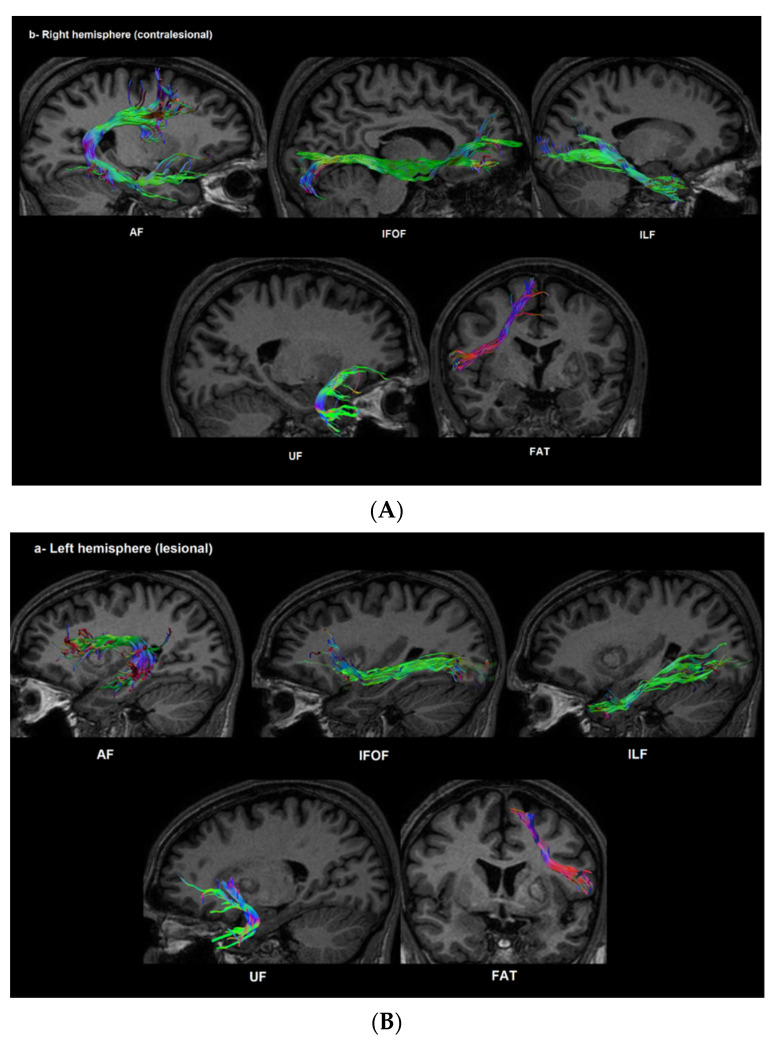
An example of the dissected white matter tracts at both hemispheres in a patient with post-stroke aphasia. (**A**) The dissected tracts at the left side, and (**B**) the dissected tracts at the right side. AF; arcuate fasciculus, IFOF; inferior fronto-orbital fasciculus, ILF; inferior longitudinal fasciculus, UF; uncinate fasciculus, FAT; frontal aslant tract.

Dissection of the AF, FAT, ILF, UF and the inferior fronto-occipital tract (IFOF) was performed, using a two ROI approach [33]. An example of the dissected tracts of one subject is shown in Figure 3. Tract averages of the diffusion measures were then extracted for each of the dissected tracts.

### 2.5. Statistical Analysis

Data analysis was performed using the IBM SPSS statistical software Package, version 23. Comparison between pre and post session data for HSS rating scales as well as comparison of both FA and MD of the dissected tracts bilaterally between pre- and post-tDCS treatments were performed using paired Student’s *t*-test. Two-way ANOVA repeated measure analysis with the main factor time (pre and post sessions) X group (real versus sham group) was performed for the assessment of the total HSS and the different components of the scale. To correct for multiple comparisons across the two diffusion measures (FA and MD), the significant threshold was set to *p* < 0.025 according to the Bonferroni correction. Partial correlation between ΔFA and ΔMD (for measures that showed significant changes after treatment) and ΔHSS (in patients performed MRI) was calculated while controlling for the effect of lesion volume. *p* < 0.05 was considered significant.

## 3. Results

### 3.1. Demographic and Clinical Data

Demographics and clinical data of all patients at baseline are illustrated in Table 1. Individual data of each participant, including the demographic and clinical assessment, are illustrated in Table 2. All patients tolerated and completed ten sessions of tDCS with no evidence of side effects. Patients receiving real tDCS showed significant improvements in the total and different components of HSS after completing all tDCS sessions (pre- versus post-sessions using paired *t*-test). In the sham group no significant improvement was recorded either in the total or in the different components of the HSS scores. Two-way ANOVA repeated measure analysis showed significant interaction between group (real and sham group) X time (pre- and post-sessions) revealing significant improvement in the experimental group particularly in total HSS, repetition, and fluency, which were absent in the control group receiving sham tDCS (cf. Table 3).

### 3.2. Lesion Overlay Map for the Experimental Group Receiving tDCS

**Figure 4:**
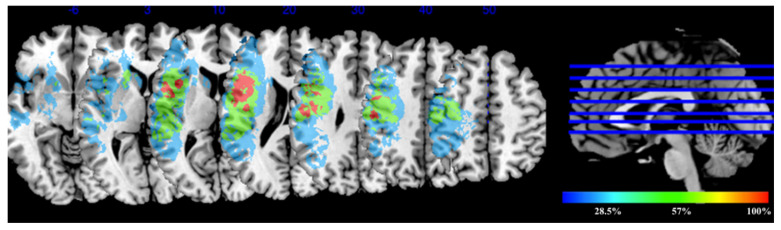
Lesion overlay map: the color bar indicates the degree of lesions overlap. Regions with the highest overlap are shown in red color (100%) and regions with the least overlap are shown in blue (28.5%).

Lesion volumes: range = 3–78 cm^3^, mean = 33 ± 27.8. The lesion overlay map (Figure 4) shows heterogeneous distribution of lesion locations among the patients. The areas with least lesion overlap are shown in blue and represented 28.5%, while areas with the highest lesion overlap are shown in red. Those with the highest overlap percentage represented all individuals (100%), and were found in the left inferior frontal operculum, rolandic operculum, and left insula.

### 3.3. Diffusion Data

Non-reconstruction of the left AF was encountered in one patient, due to the severe damage of the tract. Otherwise all other tracts at both sides were successfully reconstructed.

**Figure 5:**
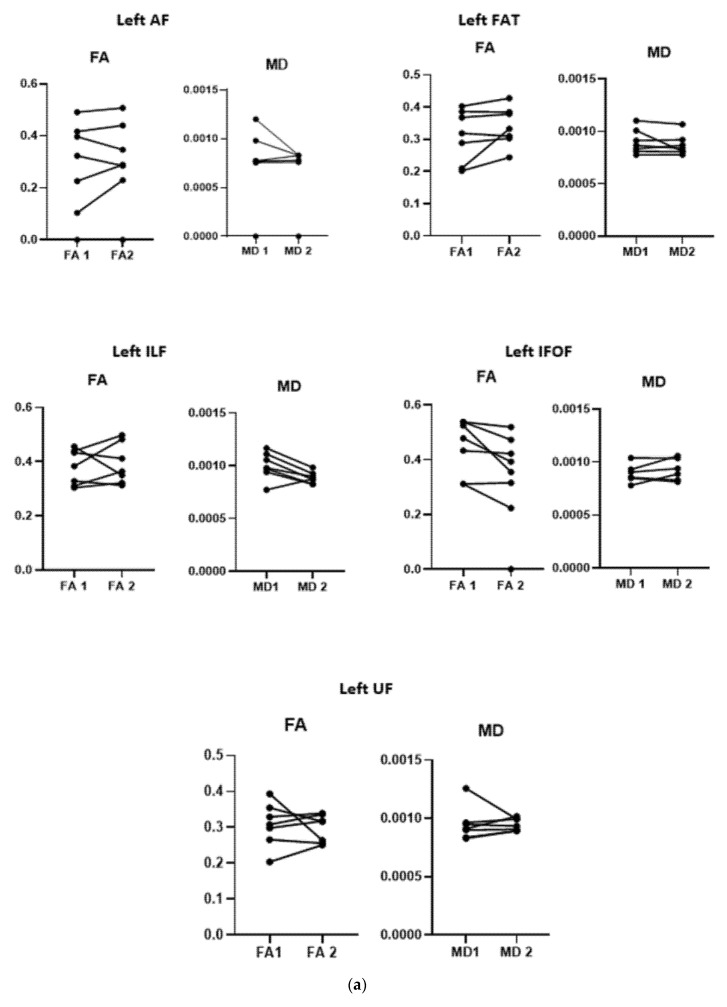

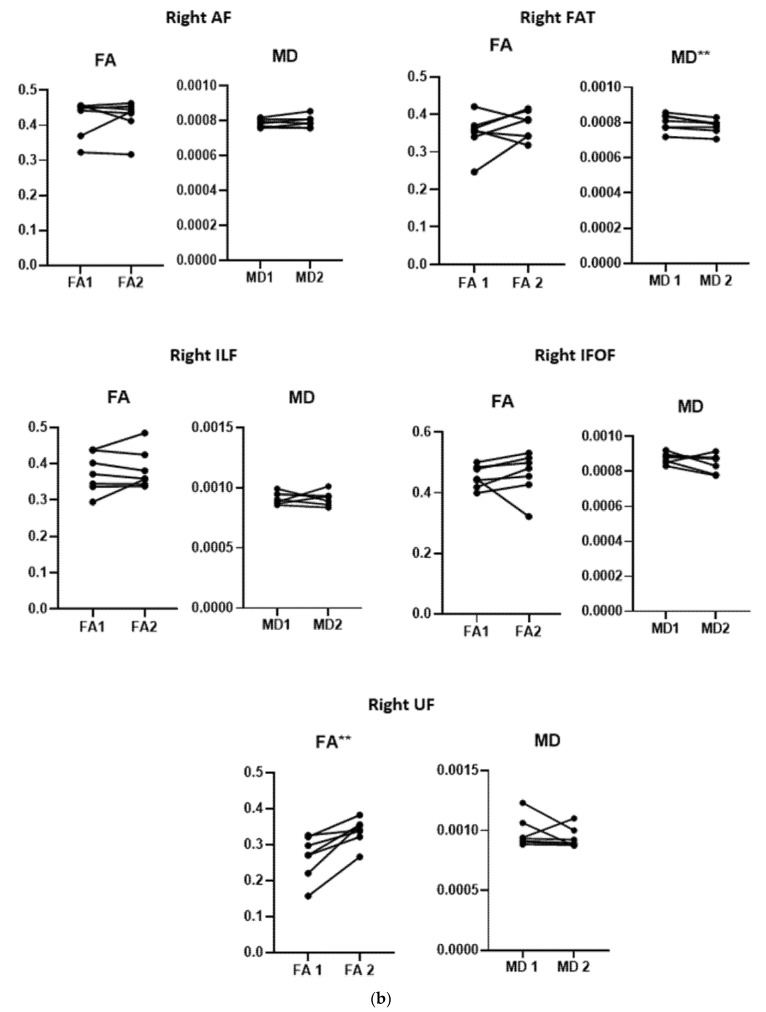
(**a**) The mean of the diffusion measures (FA and MD) of the dissected white matter tracts before (1) and after (2) tDCS treatment at the left side. AF: arcuate fasciculus; IFOF: inferior fronto-orbital fasciculus; ILF: inferior longitudinal fasciculus; UF: uncinate fasciculus; FAT: frontal aslant tract; FA: fraction anisotropy; MD: mean diffusivity. ** indicate significant changes. (**b**) The mean of the diffusion measures (FA and MD) of the dissected white matter tracts before (1) and after (2) tDCS treatment at the right side. AF: arcuate fasciculus; IFOF: inferior fronto-orbital fasciculus; ILF: inferior longitudinal fasciculus; UF: uncinate fasciculus; FAT: frontal aslant tract; FA: fraction anisotropy; MD: mean diffusivity. ** indicate significant changes.

There was a significant increase in the FA of the right UF (*p* = 0.004) and a significant reduction in the MD of the right FAT (*p* = 0.013) after tDCS treatment compared to those pre-treatment (Figure 5). No significant changes were evident in any of the diffusion measures of the other tracts in both hemispheres (Figure 5a,b).

### 3.4. Correlation between Changes in DTI Measures and Behavioral Improvement

There was a significant correlation between mean Δ FA of the right UF and improvement in fluency (r = −0.811, *p* = 0.049). There were no other significant correlations (Table 4).

## 4. Discussion

The main aim of this study was to utilize FA and MD derived from the diffusion tensor and evaluate white matter microstructural changes associated with aphasia recovery following tDCS treatment in the subacute stage. Our computational simulation shows in line with previous electrical current distribution models that electrical current flows through white matter tracts cf. [22]. Therefore, it is important to investigate the relation between white matter change and the effects of tDCS on language therapy. Anodal tDCS was applied to enhance recovery of the left hemisphere whereas cathodal tDCS was applied to suppress the maladaptive plasticity of the right hemisphere. The main effects observed were a significant increase in FA of the right UF (*p* = 0.004) and reduced MD of the right FAT (*p* = 0.013). The changes in FA of the UF was significantly correlated with improvement of fluency (r = −0.811, *p* = 0.049). This increase in the FA and the reduction in the MD of the right UF and right FAT, despite lacking specificity, may reflect thicker myelin or more densely packed axons [18], indicating neuroplasticity changes occurring in parallel with functional recovery. In agreement with our analysis, the increases in FA along with reduced MD occur during WM maturation in children [34].

The UF has been assumed to play a role in language processing, particularly the semantic aspect [35]. There is some debate, however, in this respect [35,36]. Similarly, there are disagreements among studies about its lateralization [37,38]. These discrepancies perhaps relate to the fact that the UF is comprised of different subcomponents [39], each having a distinct function, with some being lateralized while others are not. Nevertheless, our results revealing a significant correlation with fluency improvement support the notion that the right UF is involved in aphasia recovery. Moreover, our data are in line with the few studies that have acknowledged the contribution of UF in speech fluency [40,41]. Previous studies suggest that the left FAT is implicated mainly in speech fluency [19,40], whereas the right one was found to be related to working memory [42] and executive function [43]. Yet, in line with our results, Chenausky et al., found that the right FAT was significantly correlated with speech fluency in 10 minimally verbal autistic participants [44]. Furthermore, since Saur et al. found that the cortical areas connected by the right FAT are involved in recovery from aphasia, particularly in the subacute stage [9], it seems likely that the right FAT can also be involved in the recovery seen in the present cases.

Unlike some reports [11,14,16,45], no significant WM microstructural changes were evident in the left side following aphasia recovery. This is probably related to the difference in post-stroke stages. In the subacute stage, aphasia recovery most likely results from the right hemispheric contribution [9]. Furthermore, despite applying anodal tDCS over the left side and cathodal tDCS over the right hemisphere, significant WM microstructural changes were evident only in the contralesional right hemisphere. The latter may result from transmission of the left excitatory impulses via inter-hemispheric connections, or perhaps suppression of maladaptive plasticity at the right side, allowing spontaneous recovery from other regions (right UF and FAT in our case) to further pronounce, as it seems as though the response from the right side is rather heterogenous. In this sense, Hope et al. revealed that hypertrophy of the right anterior temporal region was associated with good recovery, while hypertrophy of the right posterior temporal as well pre-central regions were associated with behavioral worsening [46]. In fact, very few studies investigated the effect of tDCS on aphasia at the subacute stage. In these studies, when only anodal tDCS was applied at the left inferior frontal region to both experimental and sham group, behavioral improvement was evident in both groups. Yet there was no significant difference in the improvement between the two groups [47,48], suggesting that language improvement was mainly due to spontaneous recovery. On the other hand, when bi-hemispheric (left anodal and right cathodal) tDCS or only right cathodal tDCS was applied, significant language improvement was evident in the experimental groups compared to the sham groups [49,50]. Therefore, findings from the above studies indicate that spontaneous recovery can occur at the subacute stage and that right cathodal tDCS was essential to enhance the aphasia recovery at such stage.

Consistent with our findings, a number of other reports revealed the contribution of the contralateral right hemispheric WM in the recovery from aphasia after different types of treatment. For instance, Schlaug et al. found a significant increase in volume and number of AF fibers in six right-handed aphasic patients with left hemispheric stroke, after receiving melodic intonation therapy (MIT) [13]. Such interventions were chosen to target the right hemisphere, where melodic intonation is processed. Similarly, an 11-year-old right-handed patient with left hemisphere stroke underwent MIT and showed a complete recovery from aphasia after one year. It was associated with significant increases in the volume of both right AF and UF [16]. Another study employed repetitive TMS in eight aphasic patients, showing increase in the FA in different WM regions (near and away from the stimulation site) both at the right and left hemispheres. The authors attributed these changes to inter- and intra-hemispheric connections [11]. Furthermore, Wan et al. also revealed contralesional changes in WM properties after MIT. Yet, opposed to our analysis, they reported significant reduction (rather than increase) in the FA of different WM regions corresponding to the AF. The reduction in the FA of WM underlying the right inferior frontal gyrus was associated with fluency improvement [15]. Taken all together, these results further support the heterogenous response of the contralateral hemisphere and suggest that suppression of some regions might encourage aphasia recovery. Similarly, Hope et al. suggested that suppression of the right pre-central with activation of the right anterior temporal regions might enhance recovery [4].

### Limitations

The small sample size is a major limitation of this study. Other studies conducted in this respect, however, exhibited a similar caveat (cf. 5, 7). Nevertheless, larger studies are still required to validate these results. Moreover, constrained spherical deconvolution (CSD) is better performed at higher b-value and diffusion encoding directions than those performed in the present study. Yet, CSD at b = 1000 and diffusion directions = 32 has shown to be reliable [51] and adequate to investigate white matter in clinical study [52]. This is the first preliminary pilot study to investigate WM changes underlying tDCS. Since evaluation of WM changes was not attainable for the sham arm in this study, one might argue that the observed effects could have been due to spontaneous compensatory reorganization. However, our data reveal that significant clinical improvement was only observed in the experimental group receiving real tDCS and was absent in the control group receiving sham tDCS as a placebo condition. Moreover, previous clinical studies investigating the effects of intensive language training on aphasic patients show that only in the treated group, but not the untreated group WM changes can be observed (cf. 5–9). Schlaug et al. found a significant increase in the number of AF fibers and AF volume comparing post with pre-treatment assessments in six aphasic patients receiving language therapy that could not be attributed to scan-to-scan variability [13]. Similarly, Wan et al. observed in the treated group reductions in fractional anisotropy in the WM underlying the right inferior frontal gyrus, the right posterior superior temporal gyrus, and in the right posterior cingulum. Furthermore, they found that greater improvements in speech production were associated with greater reductions in FA in the right IFG. Remarkably, these WM changes were absent in the untreated group [15]. In line with these significant correlation between fluency improvement and WM changes was also observed in our present study, providing preliminary empirical evidence for WM changes associated with aphasia recovery due to the effect tDCS. Still, we cannot be certain of the reliability of the correlation between imaging findings and language improvement due to the small sample of patients who underwent imaging. Therefore, future studies need to investigate the observed correlation in a larger sample of patients receiving tDCS. Moreover, it would be necessary to also evaluate WM changes in a sham tDCS group and compare these effects with an experimental group receiving real tDCS to causally confirm our correlative observations relating WM changes to aphasia recovery due to transcranial brain stimulation.

## 5. Conclusions

Our data support the notion that bi-hemispheric transcranial direct current stimulation (tDCS) can induce significant clinical improvement in aphasic patients, which were absent in a control group receiving sham tDCS as placebo control.

Aphasia improvement was associated with significant microstructural WM changes in the contralesional right hemisphere, following tDCS. The diffusion measures changes of the right UF and right FAT are most likely reflecting a neuroplasticity process that can improve communication along the tracts and contribute to recovery from aphasia at the subacute stage. Studies with larger sample sizes are, however, required to underpin these results. In addition, evaluation of WM changes in a sham tDCS group is also needed to rule out that these changes are not only a part of spontaneous recovery mechanism occurring at such a stage.

## Figures and Tables

**Table 1:** Details of patient’s characteristics at baseline assessment.

Features	Patients Receiving Real tDCS *n* = 12	Patients Receiving Sham tDCS *n* = 7	*p*-Value
Age mean ± SD (range)years	52.58 ± 13.12 (32–68)	53.71 ± 6.89 (45–61)	0.809
Gender			0.830
Female number (%)	4 (33.4)	2 (28.6)	
Male number (%)	8 (66.6)	5 (71.4)	
Time passed from the onset mean ± SD (range) weeks	3.08 ± 1.42 (2–6)	3.14 ± 1.77 (1–5)	0.93
Total HSS language score Mean ± SD (range)	17.08 ± 3.17 (9–20)	18.28 ± 1.60 (17–20)	0.289
Aphasia type			
Broca’s aphasia number (%)	4 (33.4)	3 (42.8)	0.678
Global aphasia number (%)	8 (66.6)	4 (57.2)	
NIHSS mean ± SD (range)	8.52 ± 4.75 (5–17)	10.0 ± 2.58 (7–13)	0.313

Note: Quantitative variables are expressed as means ± standard deviations; numbers in brackets are ranges. Qualitative variables are expressed as proportions; numbers in parentheses are percentages. *n*: number, HSS: hemispheric stroke score.

**Table 2:** Individual data of studied patients in real tDCS and sham groups.

Nr	Group	Age	Sex	Education/ Years of Education	Risk Factors	NIHSS pre	NIHSS Post	Duration Elapsed from Onset to Session Beginning	Brain Imaging	PRE Total Language HSS Score	Post 10th Session, Total Language HSS Score
1	Real	34	M	Literate/ 12 years	Rheumatic heart disease	6	1	4 weeks	Left BG and parietal and temporal operculum	14	9
2	Real	68	M	Literate/ 6 years	Hypertension, TIA	9	7	2 weeks	Left insula and frontal and temporal operculum	15	11
3	Real	58	M	Literate/ 8 years	Hypertension History of old stroke, IHD	5	2	3 weeks	Left Posterior insula, temporal and parietal operculum and post-rolandic region	18	12
4	Real	52	M	Literate/ 6 years	History of old stroke, hypertension.	9	7	3 weeks	Left Posterior insula and parietal operculum	17	12
5	Real	55	M	Illiterate/ read and write	IHD, TIA	17	15	2 weeks	Left insula and operculum	9	7
6	Real	67	M	Literate/ 6 years	hypertension	5	2	2 weeks	Left pre-rolandic region	18	11
7	Real	56	F	Illiterate	Hypertension DM, history of old stroke	17	14	4 weeks	Left full MCA territory	20	18
8	Real	65	F	Illiterate	Hypertension old stroke IHD	11	11	6 weeks	Left insula, frontal operculum and pre-rolandic region.	17	9
9	Real	32	F	Literate/ 12 years	None	5	1	4 weeks	Left full MCA territory	19	13
10	Real	32	F	Literate/ 12 years	TIA	2	0	2 weeks	Left anterior insula and frontal operculum	18	16
11	Real	60	M	Illiterate/ read and write	Hypertension, TIA	8	6	3 weeks	Left Frontal operculum, rolandic and pre-rolandic regions	20	20
12	Real	52	M	Literate/9 years	Hypertension	5	3	2 weeks	Pre-rolandic region	20	20
13	Sham	61	M	Literate/ 9 years	Hypertension smoker	7	5	5 weeks	Full MCA territory	20	18
14	Sham	45	F	Literate/ 5 years	Hypertension	7	7	1 week	BG, pre-rolandic region	17	17
15	Sham	55	M	Illiterate/ read and write	Heavy smoker	11	10	2 weeks	Full MCA territor	17	17
16	Sham	51	M	Illiterate/ read and write	Hypertension smoker	12	10	4 weeks	Insular and sub-insular region	17	17
17	Sham	61	M	Illiterate/ read and write	Hypertension smoker	8	7	5 weeks	Left Posterior insula and parietal, operculum	20	19
18	Sham	45	F	Illiterate/ read and write	NONE	12	12	1 week	Full MCA territory	20	18
19	Sham	58	M	Illiterate/ read and write	Hypertension smoker	13	11	4 weeks	Operculum, rolandic and pre-rolandic regions	17	17

IHD; ischemic heart disease, TIA; transient ischemic attack, MCA; middle cerebral artery, BG; basal ganglia; language section of HSS; language section of the Hemispheric Stroke Scale; NIHSS; The National Institutes of Health Stroke Scale.

**Table 3:** Difference in HSS language score pre and post tDCS sessions.

Experimental Group (Real tDCS) Post Session *p*-Value				Control Group (Sham tDCS)			
Rating Scales	Pre-Session	Post-Session	*p*-Value	Pre-Session	Post-Session	*p*-Value	Two-Way ANOVA Repeated Measure Analysis Time X Groups
Total HSS language score	17.1 ± 3.2 (9–20)	13.2 ± 4.4 (7–19)	0.0001 *	18.3 ± 1.6 (17–20)	17.6 ± 0.79 (17–18)	0.09	F = 9.1, df = 1 (17) 0.008 *p* = 0.02
Comprehension score	3.4 ± 2.1 (0–5)	2.3 ± 1.8 (0–5)	0.005 *	3.7 ± 1.6 (2–5)	3.4 ± 1.4 (2–5)	0.172	F = 3.3, df = 1 (17) 0.086
Naming score	4.2 ± 1.4 (0–5)	3.3 ± 1.6 (0–5)	0.005 *	4.6 ± 1.1 (2–5)	4.3 ± 1.1 (2–5)	0.178	F = 2.1, df = 1 (17) 0.107
Repetition score	4.7 ± 0.5 (4–5)	3.6 ± 1.2 (2–5)	0.002 *	5 ± 0 (5–5)	4.9 ± 0.38 (4–5)	0.356	F = 6.8, df = 1 (17) *p* = 0.018 0
Fluency score	4.8 ± 0.6 (3–5)	3.9 ± 1.2 (2–5)	0.017 *	5.0 ± 0 (5–5)	5.0 ± 0.0 (3–5)	1.0	F = 4.5, df = 1 (17) *p* = 0.049

Note: Quantitative variables are expressed as means ± standard deviations; numbers in brackets are ranges. HSS: hemispheric stroke score; tDCS: transcranial direct current stimulation. * indicate significant changes.

**Table 4:** Partial correlation between changes in diffusion measures showing significant changes after tDCS and changes in the different components of the HSS scores after tDCS.

ΔFA and ΔMD (Indicating Significant Changes after tDCS Sessions)	ΔHSS Subsets			
	Comprehension	Naming	Repetition	Fluency
Mean ΔFA of the right UF	*r* = −0.800, *p* = 0.056	*r* = −0.241, *p* = 0.645	*r* = −0.254, *p* = 0.627	*r* = −0.811, *p* = 0.049 *
Mean ΔMD of the right FAT	*r* = 0.334, *p* = 0.518	*r* = −0.461, *p* = 0.357	*r* = −0.333, *p* = 0.519	*r* = −0.480, *p* = 0.336

Note: HSS: hemispheric stroke scale, UF; uncinate fasciculus, FAT; frontal aslant tract; FA; fraction anisotropy, MD; mean diffusivity. ΔFA and ΔMD: delta diffusion measures between pre- and post-treatment. ΔHSS: delta HSS between pre- and post-treatment. * indicate significant changes.

## Data Availability

The data presented in this study are available on request from the corresponding author.
